# In Women with Previous Pregnancy Hypertension, Levels of Cardiovascular Risk Biomarkers May Be Modulated by Haptoglobin Polymorphism

**DOI:** 10.1155/2014/361727

**Published:** 2014-07-02

**Authors:** Andreia Matos, Alda Pereira da Silva, Maria Clara Bicho, Conceição Afonso, Maria José Areias, Irene Rebelo, Manuel Bicho

**Affiliations:** ^1^Genetics Laboratory, Lisbon Medical School, University of Lisbon, 1649-028 Lisbon, Portugal; ^2^Júlio Diniz Maternity, Maria Pia Hospital, 4050-371 Porto, Portugal; ^3^Department of Biochemistry, Faculty of Pharmacy and Institute for Molecular and Cell Biology, University of Porto, 4050-313 Porto, Portugal; ^4^Rocha Cabral Institute, 1250-047 Lisbon, Portugal

## Abstract

Preeclampsia (PE) may affect the risk for future cardiovascular disease. Haptoglobin (Hp), an acute phase protein with functional genetic polymorphism, synthesized in the hepatocyte and in many peripheral tissues secondary of oxidative stress of PE, may modulate that risk through the antioxidant, angiogenic, and anti-inflammatory differential effects of their genotypes. We performed a prospective study in 352 women aged 35 ± 5.48 years, which 165 had previous PE, 2 to 16 years ago. We studied demographic, anthropometric, and haemodynamic biomarkers such as C-reactive protein (CRP), myeloperoxidase (MPO), and nitric oxide metabolites (total and nitrites), and others associated with liver function (AST and ALT) and lipid profile (total LDL and cholesterol HDL, non-HDL, and apolipoproteins A and B). Finally, we study the influence of Hp genetic polymorphism on all these biomarkers and as a predisposing factor for PE and its remote cardiovascular disease prognosis. Previously preeclamptic women either hypertensive or normotensive presented significant differences in those risk biomarkers (MPO, nitrites, and ALT), whose variation may be modulated by Hp 1/2 functional genetic polymorphism. The history of PE may be relevant, in association with these biomarkers to the cardiovascular risk in premenopausal women.

## 1. Introduction 

Maternal hypertensive disorders are the most common complications of pregnancy. Pregnancy may be complicated by four distinct forms of hypertension: preeclampsia/eclampsia, chronic hypertension, preeclampsia superimposed on chronic hypertension, and gestational hypertension [1]. Arterial hypertension may be associated with inflammatory and oxidative stress. Preeclampsia as other forms of hypertensive conditions during pregnancy may affect the risk for future cardiovascular disease [2, 3].

Several authors described association between maternal pregnancy complications as preeclampsia—with greater future risk of mother to develop hypertension and atherosclerosis [2, 3]. Indeed, there are biomarkers associated with inflammatory process and blood pressure, which may lead to the future evolution of hypertensive disease of pregnancy and cardiovascular risk in women who previously developed hypertension during pregnancy [4, 5].

Haptoglobin (Hp) is an acute phase *α*2 plasma glycoprotein, synthesized in the hepatocyte and other peripheral tissues, which scavenge free haemoglobin and may modulate differentially cardiovascular risk through its antioxidant and anti-inflammatory different capacities associated with their genotypes [6, 7]. The Hp gene is expressed primarily in hepatocytes but also locally in other tissues or in cells related with inflammatory processes, such as neutrophils [8]. This protein has a pronounced anti-inflammatory action and has high affinity to a specific receptor (CD163) located in circulating monocytes, resident macrophages (M2 type), and liver Kupffer cells [9–11]. The cellular expression of this pathway of Hp, CD163 and hemoxygenase (HO-1), is strongly activated directly or indirectly by cytokines, such as interleukins (IL-6, IL-1), tumor necrosis factor alfa (TNF-*α*), growth factors (M-CSF) [12], or hormones such as catecholamines and glucocorticoids [13].

Hp may have a role in the pregnant women with hypertension playing a protection role from further cardiovascular risk, once it prevents the formation of free radicals and its accumulation in endothelial cells, catalysed by heme, therefore preventing vessel injury [9, 11, 13].

Hp has a genetic polymorphism (Hp 1.1, 2.1 e 2.2) contributing to the great variability in anti-inflammatory responses; namely, Hp 2.2 phenotype is associated with a lower antioxidant capacity than the other two Hp phenotypes because of its higher molecular mass that restricts its extra vascular diffusion [6, 7, 14]. Also the Hp 2.2/Hb complex scavenges more nitric oxide (NO) than Hp 1.1/Hb due to its longer half-life in circulation [7, 15, 16].

The inhibitory effects on prostaglandin synthesis of Hp 2.2 and Hp 2.1 are less pronounced than those of Hp 1.1 contributing differently for its lower anti-inflammatory action [6, 17, 18]. However, Hp 2.2 is the most angiogenic form in the course of chronic inflammatory processes leading to greater ischemic tissue reparation and promoting of collateral vessel formation than the other two forms [19, 20].

The *α*-chain of haptoglobin and haptoglobin-related protein (Hpr), belonging to the cluster of Hp in chromosome 16, contains a hydrophobic signal peptide that may explain its association with lipoprotein particles (HDL) or membranes [21].

The objectives of the present work were to evaluate in women with history of hypertension in pregnancy/preeclampsia the susceptibility to develop hypertension in the future and the possible relationship with Hp phenotypes; the second objective was to evaluate the influence of the Hp genetic polymorphism on circulating cardiovascular risk biomarkers and the level of blood pressure in a prospective cohort.

## 2. Materials and Methods

### 2.1. Sample Population

We studied 352 women aged 35 ± 5.48 years, and from these, 165 had preeclampsia 2 to 16 (± 6.6) years ago, which was identified from medical records at the Department of Obstetrics and Gynecology from the Júlio Diniz Maternity, Maria Pia Hospital, OPorto. The diagnosis of preeclampsia was based on criteria of the International Society for the Study of Hypertension in Pregnancy (ISSHP) [22]. Women of the control group of the same Hospital were matched for age within group on the study and similarly to the study group. They were firstly interviewed by phone. Then, they were invited to come to the research center during the same phase of their menstrual cycles for sample collection. We also evaluated some unhealthy behaviors as smoke and alcoholic habits through a questionnaire that determined women who smoked or drank after pregnancy, respectively.

Women were stratified accordingly to the criteria of the ISSHP [22] in preeclamptic (PE) and normal blood pressure in pregnancy (NBPP); in hypertensive after pregnancy (HTA), and normotensive after pregnancy (NBP), based on the criteria of European Society of Hypertension (ESH) and European Society of Cardiology (ESC) [23].

### 2.2. Haptoglobin Polymorphism Detection

**Figure 1:**
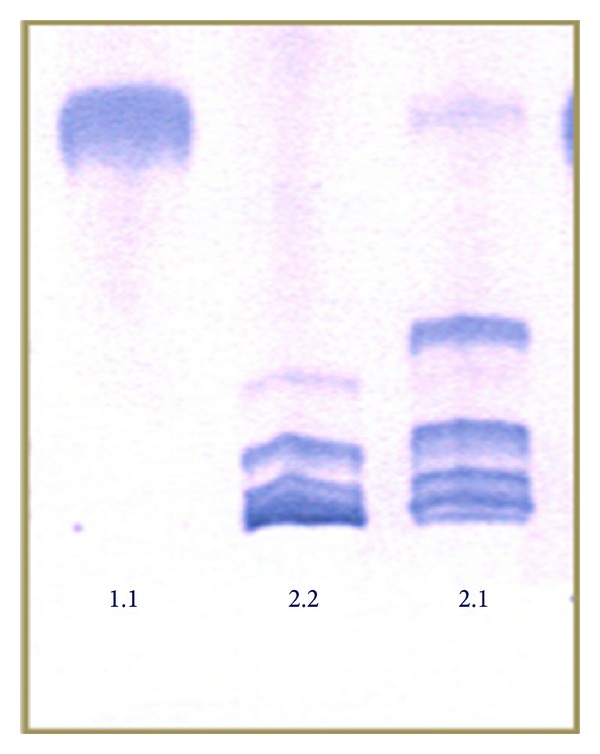
Polyacrylamide gel electrophoresis (PAGE) of Hp showing the typical pattern of bands of 1.1, 2.1, and 2.2 phenotypes.

The three phenotypes of Hp (1.1, 2.1 and 2.2) were separated from plasma using polyacrylamide gel electrophoresis (PAGE) and its presence was detected by the peroxidase activity of the complex haptoglobin—haemoglobin over the colour using substrate of o-dianisidine (Figure 1) [24, 25].

### 2.3. Circulating Cardiovascular Risk Biomarkers Determination

The different circulating biomarkers were determined by enzyme-linked immunosorbent assay (ELISA—R&D Systems Inc.) such as myeloperoxidase (MPO, ng/mL). Nitric oxide metabolites (NOx, mmol/L) and nitrites (*μ*mol/L), transaminases—AST (Aspartate transaminase, UI/L), and ALT (alanine transaminase, UI/L) were determined by conventional standardized methods. Classical biomarkers as serum lipids and lipoproteins: total cholesterol (t-cholesterol, mg/dl) and HDL and LDL cholesterol, were measured by using automated enzymatic assays (ABX Diagnostic) and apolipoprotein A and B (Apo A and B, mg/dL) by using automated immunoturbidimetric assays (ABX Diagnostic). Serum C-reactive protein (CRP, mg/L) was assessed by an immunoenzymatic method (adaptation of the method of Highton and Hessian, 1984 [26].

### 2.4. Blood Pressure and Anthropometric Parameters Evaluation

Blood pressure in mmHg (BP) was measured by an oscillometric method. Anthropometric parameters such as BMI (body mass index, Kg/m^2^) and hip (cm) and waist circumference (WC, cm) were evaluated using classic measurement instruments.

### 2.5. Statistics Analysis

In statistical analyses, we included departure from normality according to Kolmogorov Smirnov test and then adequate parametric or nonparametric tests to compare means. We also performed the Chi-square, and for pairwise comparisons between groups we used Student's *t* test or Mann-Whitney *U* test, with a probability value of <0.05 considered statistically significant. For this analysis, we used 21 version SPSS programme.

## 3. Results 

The results are shown in two parts. The first one considers the risk of preeclampsia in accordance with Hp phenotype distribution in women during pregnancy (Study 1). The second one observes the susceptibility of cardiovascular risk in women with previous preeclampsia, considering also the influence of circulating cardiovascular risk biomarkers, and Hp phenotype, in a follow-up subsample of 2 to 16 years (Study 2) (Figure 1).

*Study 1: Haptoglobin polymorphism and susceptibility for the development of preeclampsia.*

Table 1 shows distribution of Hp phenotypes in a population of normotensive (normal blood pressure in pregnancy—NBPP) and hypertensive (Preeclampsia—PE) pregnant women (*N* = 265). The NT women were significantly younger (27.93 ± 4.91, mean ± S.D.) than women with preeclampsia (29.71 ± 5.97, mean ± S.D.) (*P* = 0.011). Most women have over 34 weeks of gestation, independently of hypertension degree, but before or 34 weeks of gestation there were more significantly preeclamptic women (30.3%) (*P* < 0.001) (data not shown).

In our population of 265 Caucasian pregnant women and concerning the Hp phenotype distribution, we found no statistical differences of Hp phenotype distribution (1.1, 2.1, and 2.2) between 128 normotensive women (NT) and 137 PE (*P* = 0.421) (Table 1).

We also evaluated the distribution of Hp phenotype in all preeclamptic women at age of diagnosis between** ≤ **34 weeks of gestation and > 34 weeks of gestation and we observed no significant differences (Table 2).

*Study 2: The susceptibility of cardiovascular risk in women with previous preeclampsia and the influence of risk biomarkers and its modulation by the Hp phenotype at long term (2–16 years).*

In the follow-up group we evaluated anthropometric and hemodynamic parameters and some biomarkers of cardiovascular risk in a sample of previously preeclamptic women and compared them with normotensive ones adjusted for age at pregnancy. We also study the influence of the Hp phenotype on the levels of biomarkers in circulation.

### 3.1. Anthropometric and Hemodynamic Parameters

This sample consisted of 150 women aged 20 to 35 years old (min.: 20–max: 47; 35.24 ± 5.48 (mean ± S.D.) and minimum BMI of 17.1 (underweight) to 42.7 (obesity) (26.39 ± 4.57 Kg/m^2^, mean ± S.D.), who were recruited for this prospective study, 2–16 years after delivery. During pregnancy, 60 women were NT and 90 were preeclamptic. In this group, 16.2% have smoke habits and 4.7% consume alcoholic beverages, after pregnancy.

In this sample, when evaluating the values of blood pressure and anthropometric data we observed significantly mean higher values in previously preeclamptic women (PE) for BMI (27.05 ± 4.79, *P* = 0.033), WC (89.54 ± 15.64, *P* = 0.004), systolic blood pressure (134.99 ± 16.50, *P* < 0.001), and diastolic blood pressure (85.93 ± 18.28, *P* < 0.001), when compared with NBPP (Table 3).

### 3.2. Cardiovascular Risk Circulating Biomarkers

In order to evaluate biochemical biomarkers potentially implicated in cardiovascular risk, we found statistically significant differences with higher concentrations for previously PE comparing with NBPP, for MPO (85.67 ± 39.39, *P* = 0.020), nitrites (19.12 ± 7.01, *P* < 0.001), ALT (19.00 ± 1.36, *P* = 0.003), and Apo B (0.64 ± 0.14, *P* = 0.023) (Table 4) and slightly higher values for NOx (99.44 ± 39.52, *P* = 0.061).

According to classification during pregnancy [22] and considering the Hp phenotype, we found a variation in anthropometric characteristics and blood pressure and also in the cardiovascular risk biomarkers, classical or not between normotensive and preeclamptic women (Table 5). In women with Hp 1.1 and 2.1 phenotypes, we found significantly higher values in preeclamptic women (PE) in WC (90.78 ± 17.58), systolic and diastolic blood pressures (134.65 ± 18.31 and 86.19 ± 19.42, *P* < 0.001), MPO (94.17 ± 42.14, *P* = 0.008), nitrites (19.98 ± 8.53, *P* < 0.001), ALT (19.98 ± 8.53, *P* = 0.005), and Apo A (0.98 ± 0.16, *P* = 0.011) and also a trend in BMI (26.95 ± 5.46, *P* = 0.061) compared with normotensive ones (Table 5).

On the other hand, for Hp 2.2 phenotype we found also significant differences with higher levels in preeclamptic women, for systolic and diastolic blood pressures (135.61 ± 12.79 and 85.45 ± 16.26, *P* < 0.001) and nitrites (18.01 ± 4.44, *P* = 0.007) compared with normotensive ones (Table 5).

When comparing Hp phenotypes subgroups (1.1 plus 2.1 versus 2.2), within either NBPP or PE groups, we found significant differences as follows: higher values of Apo A (0.90 ± 0.17 versus 1.07 ± 0.22, *P* = 0.002) and CRP (0.50 ± 0.10 versus 0.70 ± 0.09, *P* = 0.026) associated with Hp 2.2, in NBPP and PE groups, respectively (data not shown).

**Figure 2:**
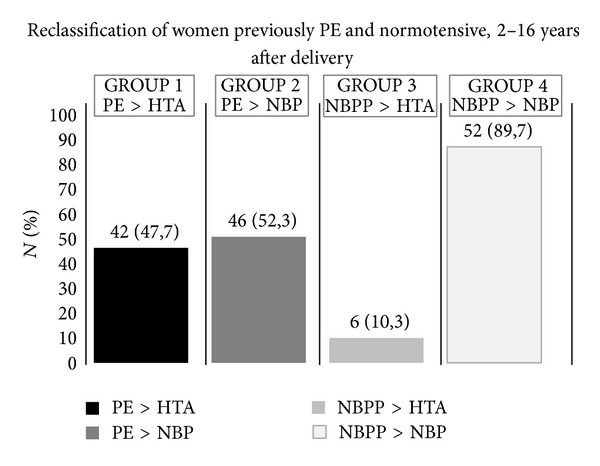
Reclassification of women previously PE and normotensive, 2–16 years after delivery. This reclassification took into account the definitions of hypertension according to diastolic and/or systolic pressures during pregnancy and 2–16 years after pregnancy and childbirth. Preeclamptic (PE), normal blood pressure in pregnancy (NBPP), hypertensive after pregnancy (HTA), and normotensive after pregnancy (NBP).

Women after pregnancy were then stratified accordingly to previously preeclamptic (PE) or normotensive (NBP) women corresponding to reclassifying by the criteria of the ESH/ESC [23]. We found that 47.7% of preeclamptic women developed hypertension (Group 1) and that only 10.3% of normotensive women during pregnancy developed hypertension afterwards, Group 3 as in shown in Figure 2 (*P* < 0.001). Two other groups of women, such as Group 2 of previously preeclamptic women that became normotensive and Group 4 of previously normotensive women that maintain normotensive, were analysed (Figure 2).

When we evaluated circulating cardiovascular risk biomarkers, we found that preeclamptic women that subsequently became normotensive (Group 2, PE > NBP) have some clear characteristics of the hypertensive subjects (Group 1, PE > HTA), namely, BMI, WC, pulse pressure, CRP, MPO, nitrites, nitric oxide total metabolites (NOx), transaminases, and lipid profile (Table 6). Moreover the preeclamptic women that developed hypertension were significantly older than the preeclamptic women that did not develop hypertension (Group 1, PE > HTA versus Group 2, PE > NBP) (36.64 ± 5.16 versus 33.50 ± 5.29, *P* = 0.008). The Groups 2 and 3 only differ significantly in systolic and diastolic blood pressures with higher levels for Group 3 (140.00 ± 6.23 and 86.00 ± 5.87, *P* < 0.001 and *P* = 0.008, resp.), and for nitrites with higher levels in Group 2 (18.02 ± 3.89, *P* < 0.001) (Table 6).

The pure normotensive group or Group 4 (NBPP > NBP) differs significantly in BMI (28.71 ± 5.11, *P* = 0.033), systolic (140.00 ± 6.23, *P* < 0.001), diastolic blood pressures (86.00 ± 5.87, *P* < 0.001), and pulse pressure (54.00 ± 9.53, *P* = 0.040) and slightly in CRP (0.70 ± 0.24, *P* = 0.055), when comparing with Group 3, with higher values for the this one (Table 6). When comparing this group (Group 4, NBPP > NBP) with women that became normotensive after a preeclamptic episode (Group 2, PE > NBPP) we found significant differences in BMI (26.85 ± 4.69, *P* = 0.033), WC (88.38 ± 12.01, *P* = 0.005), systolic blood pressure (125.04 ± 8.85, *P* < 0.001), pulse pressure (50.96 ± 8.38, *P* = 0.004), MPO (86.49 ± 44.39, *P* = 0.032), nitrites, (18.02 ± 3.89, *P* > 0.001), ALT (19.00 ± 2.07, *P* = 0.021), and Apo B (0.65 ± 0.13, *P* = 0.040), with higher values for Group 2 (PE > NBP).

Extreme groups (Group 1—PE > HTA and Group 4—NBPP > NBP) differ significantly with higher levels for Group 1 in BMI (27.22 ± 5.00, *P* = 0.016), WC (90.56 ± 18.96, *P* = 0.010), systolic (145.88 ± 16.11, *P* < 0.001), diastolic (98.90 ± 16.26, *P* < 0.001) blood pressures, MPO (82.74 ± 11.04, *P* = 0.010), nitrites (23.04 ± 13.07, *P* = 0.037), and ALT (19.00 ± 1.85, *P* = 0.031) (Table 6).

We evaluated the distribution of the Hp phenotypes among the four subgroups and we did not find differences between them (*P* = 0.273), even within subgroups of previously preeclamptic or normotensive women considering separately (*P* = 0.130 and 0.185, resp.) (Table 7).

In order to study the influence of the Hp phenotypes (1.1 plus 2.1 versus 2.2) in cardiovascular risk, we analyse in these newly identified four groups the levels of biomarkers and their variation according to Hp phenotype (see Supplementary table in Supplementary Material available online at http://dx.doi.org/10.1155/2014/361727). Relative to individual groups, we found significant differences only in Group 4 (NBPP > NBP, previously normotensive pregnant women that maintain normotensive) with higher levels of Apo A (0.89 ± 0.17 versus 1.07 ± 0.23, *P* = 0.003) and slightly elevated differences for HDL (49.00 ± 1.46 versus 54.20 ± 2.04, *P* = 0.068) associated with Hp 2.2 phenotype.

Considering only the Hp 1.1 plus 2.1 phenotypes, we observed between Groups 1 and 2 (PE > HTA versus PE > NBP) differences for HDL cholesterol with higher values at Group 2 (46.00 ± 1.69 versus 53.00 ± 1.39, *P* = 0.053), and between Groups 2 and 3 (PE > NBP versus NBPP > HTA) we found differences in nitrites (17.90 ± 2.89 versus 9.00 ± 0.00, *P* < 0.001) with higher values for PE > NBP, and between Groups 3 and 4 (NBPP > HTA versus NBPP > NBP) differences were found for BMI (28.71 ± 5.11 versus 24.45 ± 3.22, *P* = 0.010), systolic blood pressure (140.00 ± 6.23 versus 115.50 ± 11.06, *P* < 0.001), diastolic blood pressure (86.00 ± 5.87 versus 70.47 ± 10.26, *P* = 0.001), pulse pressure (54.00 ± 9.53 versus 45.03 ± 7.12, *P* = 0.011), and CRP (0.70 ± 0.24 versus 0.30 ± 0.17, *P* = 0.029), but for WC (89.00 ± 15.11 versus 81.20 ± 8.48, *P* = 0.078) differences were slight. Between Groups 2 and 4 (PE > NBP versus NBPP > NBP) we found significantly mean higher levels for BMI (26.88 ± 5.23 versus 24.45 ± 3.44, *P* = 0.026), WC (88.93 ± 12.51 versus 24.45 ± 3.22, *P* = 0.005), systolic blood pressure (124.56 ± 9.02 versus 115.50 ± 11.06, *P* = 0.001), MPO (96.93 ± 45.84 versus 54.38 ± 30.75, *P* = 0.009), nitrites (17.90 ± 2.89 versus 8.99 ± 2.32, *P* < 0.001), Apo A (0.99 ± 0.15 versus 0.89 ± 0.17, *P* = 0.011), and ALT (18.00 ± 1.65 versus 15.00 ± 1.34, *P* = 0.025). Finally for extreme Groups 1 and 4 (PE > HTA versus NBPP > NBP) there were significant differences in WC (92.74 ± 22.68 versus 81.20 ± 8.47, *P* = 0.022), systolic blood pressure (147.56 ± 19.17 versus 115.50 ± 11.06, *P* < 0.001), diastolic blood pressure (100.92 ± 18.48 versus 70.47± 10.26, *P* < 0.001), and MPO (80.33 ± 6.42 versus 54.38 ± 30.75, *P* = 0.014), as well as a trend in BMI (29.97 ± 5.93 versus 24.45 ± 3.22, *P* = 0.063) and ALT (0.45 ± 0.19 versus 0.30 ± 0.17, *P* = 0.055) (supplementary table).

By other hand, when consider only the Hp 2.2 phenotype, we obtained differences between Groups 1 and 2 (PE > HTA versus PE > NBP) with higher values for systolic (143.41 ± 10.12 versus 126.14 ± 8.66, *P* < 0.001) and diastolic (95.94 ± 12.24 versus 72.71 ± 10.37, *P* < 0.001) blood pressures. Between Groups 1 and 4 (PE > HTA versus NBPP > NBP) we found differences in nitrites with higher values in Group 1 (17.53 ± 1.89 versus 11.54 ± 5.06, *P* = 0.052) (data not shown).

## 4. Discussion

Cardiovascular disease in pre- and postmenopausal women is the most prevalent cause of morbidity including metabolic syndrome with abdominal obesity, dyslipidaemia, insulin resistance, and hypertension.

In the last 10 years, several studies demonstrate that history of preeclampsia increases the risk for development of cardiovascular disease [2, 5]. Hypertensive disease of pregnancy in particular preeclampsia (PE) is characterized by a proinflammatory state of low intensity initiated in the placenta after under-perfusion, hypoxia, and local oxidative stress. This state leads to endothelial dysfunction and secondarily the clinical symptoms of PE [27]. The initial phenomena of ischemia reperfusion of placenta give places probability to the formation and release of advanced glycation end products (AGEs) that secondarily activates the AGE-RAGE (receptor of AGE) axis [28, 29].

AGE-RAGE axis activates an acute phase response locally in placenta or systemically in liver where one of its the components is haptoglobin (Hp) that initiates the axis Hp-CD163-heme oxygenase (HO) that leads to the switch of Th1 to Th2 of acquired immune response [12, 20, 30].

In our present study we did not observe a clear association of the Hp phenotypes with susceptibility to preeclampsia or to its long-term prognosis. But the presence of Hp allele 1 seems to be a protective factor for these outcomes, as it was observed by the other authors [31–33]. For some authors, this can be due to the great immune tolerance potential of the Hp 2.1 phenotype [34, 35]. However, this subject is controversial [36, 37]. The early PE, more characteristics of placenta dysfunction versus late PE, linked to endothelial dysfunction due to constitutional factors such as body mass index (BMI) and metabolic syndrome, cannot be explained by Hp polymorphism (Table 2).

In our cohort, we observed independently of age, significant higher BMI, WC, and systolic and diastolic blood pressure in previously preeclamptic women. The same happens for more elevated MPO, nitrites, ALT, and Apo B concentrations in blood. These results are in accordance with those of other authors [3, 38].

When we analysed those biomarkers (anthropometric, haemodynamic, and circulatory) stratified by Hp phenotypes (Hp 1.1 plus 2.1 versus 2.2), we found significant differences between previously PE versus normotensive (Table 5), respectively, for WC, MPO, ALT, and Apo A (more elevated in carriers of Hp 1.1 plus 2.1 phenotypes). For lipid profile biomarkers, Hp 2.2 in both NBPP and PE groups has higher values than Hp allele 1 carriers. This can be explained by great expression of Apo A in oxidative condition [21]. Elevated MPO probably is related to NO bioavailability through its oxidation into nitrites, which were also more elevated in previously PE women of both Hp phenotypes [39]. MPO free in plasma or serum represents that one which is mobilized from the vessel wall to the lumen affecting NO bioavailability [40]. After reclassification according to actual blood pressure of previously PE women, in two groups with (Group 1) or without (Group 2) actual hypertension and using the same criteria for previously normotensive women we could have a more real picture of risk of the women having hypertensive disease, years after pregnancy and the natural history of cardiovascular disease in premenopausal women (Figure 2). Between the two subgroups of previously PE women there is a difference in age, with a mean age lower in NBP (Table 6). These women probably became hypertensive later. The same situation relative to age was observed between the two normotensive Groups 3 and 4. Group 3 seems to have characteristics of metabolic syndrome features, like WC, pulse pressure, and CRP. This situation is also observed comparing Group 4 with Group 2 (PE > NBP) and similarly comparing with Group 1 (PE > HTA) as is observed in Table 6.

Finally, haptoglobin polymorphism also did not influence apparently the natural history of previously preeclamptic and normotensive Groups 1 and 2, premenopausal one (Table 7). After our trial to clarify the influence of that polymorphism in some circulating risk biomarkers (supplementary table), in women with Hp 1 allele (Hp 1.1 plus 2.1), we observe a trend for higher values of HDL cholesterol in Group 2 (PE > NBP), compared with women PE > HTA (Group 1), even after adjusting for age.

The difference between groups previously with PE that became hypertensive (Group 1) or yet normotensive (Group 2) and also Group 3 (NBPP > HTA), as compared with Group 4 (NBPP > NBP, previously normotensive pregnant women that maintain normotensive) depends on surrogate biomarkers of metabolic syndrome and NO bioavailability, sustained by Hp 2.2 phenotype.

## 5. Conclusions 

Women with previous preeclampsia and premenopausal, even if became normotensive, presented significant differences compared with previous normotensive women during pregnancy in some classic cardiovascular risk biomarkers as well as in some others, associated with metabolic syndrome, NO bioavaibility and inflammatory process. These biomarkers variation may be modulated by haptoglobin functional genetic polymorphism more relevant in the carriers of haptoglobin 1 allele. The history of hypertensive disease in pregnancy may be relevant, in association with these biomarkers including genetic ones, to the prevention of cardiovascular disease in particular of postmenopausal women.

## Supplementary Material

In supplementary table, four distinguishes groups, which results from women that were classified in preeclamptic (PE) and normal blood pressure in pregnancy (NBPP) and reclassified in hypertensive after pregnancy (HTA) and normotensive after pregnancy (NBP), based on the criteria of European Society of Hypertension (ESH) and European Society of Cardiology (ESC). These groups were characterized in conformity of hypertension classification and anthropometric, hemodynamic, cardiovascular risk biomarkers and other biochemical parameters in Hp 1.1 plus 2.1 phenotypes.

## Figures and Tables

**Table 1:** Distribution of Hp phenotypes in women with normal blood pressure in pregnancy (NBPP) and preeclamptic women (PE).

Phenotype	NBPP *n* = 128	PE *n* = 137	*P* value
	*n* (%)	*n* (%)	
Hp 1.1	28 (21.9)	22 (16.1)	0.421
Hp 2.1	66 (51.6)	72 (52.5)	
Hp 2.2	34 (26.5)	43 (31.4)	

Chi-square test.

**Table 2:** Distribution of Hp phenotypes in the sample of preeclamptic women (PE) stratified by age of gestation at diagnosis.

	Hp 1.1 *n* = 19	Hp 2.1 *n* = 66	Hp 2.2 *n* = 42	*P* value
≤34 weeks of gestation, *n* (%)	6 (14.6)	23 (56.1)	12 (29.3)	0.791
>34 weeks of gestation, *n* (%)	13 (15.1)	43 (50.0)	30 (34.9)	

Chi-square test.

Preeclamptic women (PE) with diagnosis before 34 weeks of gestation (≤34 weeks of gestation) and after 34 weeks of gestation (>34 weeks of gestation).

**Table 3:** Comparison of anthropometric and blood pressure data in women with normal blood pressure in pregnancy (NBPP) and preeclamptic women (PE).

	NBPP *n* (mean ± SD)	PE *n* (mean ± SD)	OR	CI (95%)	*P* value^†^
Age (years)	60 (35.62 ± 5.62)	89 (34.99 ± 5.40)	0.979	(0.922–1.040)	0.492
BMI (Kg/m^2^)	59 (25.40 ± 4.05)	88 (27.05 ± 4.79)	1.090	(1.007–1.180)	**0.033**
WC (cm)	56 (82.77 ± 9.85)	88 (89.54 ± 15.64)	1.048	(1.015–1.082)	**0.004**
Systolic BP (mmHg)	58 (118.88 ± 13.38)	88 (134.99 ± 16.50)	1.095	(1.059–1.133)	**<0.001**
Diastolic BP (mmHg)	58 (72.21 ± 10.08)	88 (85.93 ± 18.28)	1.076	(1.043–1.110)	**<0.001**
Pulse pressure	58 (46.67 ± 9.30)	88 (49.06 ± 11.91)	1.021	(0.990–1.053)	0.196

^†^Values adjusted for age (regression binary logistic).

Body mass index (BMI), waist circumference (WC), systolic blood pressure (Systolic BP), diastolic blood (Diastolic BP), and pulse pressure.

**Table 4:** Comparison of cardiovascular risk biomarkers in women with normal blood pressure in pregnancy (NBPP) and women with preeclampsia (PE).

	NBPP *n* (mean ± SD)	PE *n* (mean ± SD)	*P* value
CRP (mg/L)^††^	56 (0.40 ± 0.11)	83 (0.60 ± 0.07)	0.179
MPO (ng/mL)^†^	24 (62.27 ± 30.88)	32 (85.67 ± 39.39)	**0.020**
Nitrites (*μ*mol/L)^†^	25 (10.12 ± 3.80)	32 (19.12 ± 7.01)	**<0.001**
NO_*x*_ (*μ*mol/L)^†^	25 (79.18 ± 38.06)	29 (99.44 ± 39.52)	0.061
AST (UI/L)^††^	60 (18.00 ± 0.65)	90 (19.00 ± 0.72)	0.083
ALT (UI/L)^††^	60 (15.50 ± 1.03)	90 (19.00 ± 1.36)	**0.003 **
t-Cholesterol (mg/dL)^†^	60 (206.57 ± 34.29)	90 (207.18 ± 39.33)	0.922
Non HDL cholesterol^†^	59 (157.00 ± 35.60)	90 (158.17 ± 37.96)	0.851
LDL (mg/dL)^†^	59 (138.75 ± 32.30)	90 (158.17 ± 37.96)	0.855
HDL (mg/dL)^††^	59 (50.00 ± 1.11)	90 (49.00 ± 0.89)	0.479
Apo A (mg/dL)^†^	58 (0.95 ± 0.20)	87 (0.99 ± 0.17)	0.129
Apo B (mg/dL)^†^	58 (0.59 ± 0.13)	87 (0.64 ± 0.14)	**0.023**

^†^Independent sample *t*-test; and values are means ± standard deviation (SD).

^††^Mann-Whitney *U* test; and values are median ± standard error (SE).

C-reactive protein (CRP), Myeloperoxidase (MPO), nitrites, nitric oxide metabolites (NO_*x*_), aspartate transaminase (AST), alanine transaminase (ALT), total cholesterol (t-cholesterol), non HDL cholesterol, apolipoprotein A and B (Apo B and Apo A), low density lipoprotein (LDL), and high density lipoprotein (HDL).

**Table 5:** Comparison of blood pressure, anthropometric characteristics, and classic or not biomarkers between women with normal blood pressure in pregnancy (NBPP) and preeclamptic women (PE) according to Haptoglobin phenotype.

	Hp 1.1 plus 2.1			Hp 2.2		
	NBPP *n* (mean ± SD)	PE *n* (mean ± SD)	*P* value	NBPP *n* (mean ± SD)	PE *n* (mean ± SD)	*P* value
BMI (Kg/m^2^)^†^	41 (25.18 ± 3.80)	57 (26.95 ± 5.46)	0.061	18 (25.90 ± 4.65)	31 (27.23 ± 3.32)	0.249
WC (cm)^†^	38 (82.43 ± 10.0)	57 (90.78 ± 17.58)	**0.004 **	18 (83.47 ± 9.84)	31 (87.26 ± 11.15)	0.238
Systolic BP (mmHg)^†^	40 (119.18 ± 13.67)	57 (134.65 ± 18.31)	**<0.001 **	18 (118.22 ± 13.01)	31 (135.61 ± 12.79)	**<0.001**
Diastolic BP (mmHg)^†^	40 (72.80 ± 11.18)	57 (86.19 ± 19.42)	**<0.001 **	18 (70.89 ± 7.15)	31 (85.45 ± 16.26)	**<0.001 **
Pulse pressure^†^	40 (46.38 ± 8.12)	57 (48.46 ± 11.89)	0.308	18 (47.33 ± 11.76)	31 (50.16 ± 12.07)	0.429
CRP (mg/L)^††^	38 (0.40 ± 0.15)	55 (0.50 ± 0.10)	0.697	18 (0.40 ± 0.12)	28 (0.70 ± 0.09)	0.106
MPO (ng/mL)^†^	17 (57.89 ± 32.47)	18 (94.17 ± 42.14)	**0.008 **	7 (72.89 ± 25.64)	14 (74.75 ± 33.89)	0.900
Nitrites (*μ*mol/L)^†^	18 (9.57 ± 3.19)	18 (19.98 ± 8.53)	**<0.001 **	7 (11.54 ± 5.06)	14 (18.01 ± 4.44)	**0.007 **
NO_*x*_ (*μ*mol/L)^†^	18 (79.04 ± 37.15)	17 (101.32 ± 48.79)	0.141	7 (79.53 ± 43.39)	12 (96.79 ± 22.34)	0.356
AST (UI/L)^††^	41 (18.00 ± 0.70)	59 (19.00 ± 0.66)	0.084	19 (8.00 ± 1.40)	31 (19.00 ± 1.67)	0.582
ALT (UI/L)^††^	41 (15.00 ± 1.13)	59 (18.00 ± 1.04)	**0.005 **	19 (19.00 ± 2.11)	31 (20.00 ± 3.34)	0.234
t-Cholesterol (mg/dL)^†^	41 (203.73 ± 33.24)	59 (202.66 ± 38.65)	0.886	19 (212.68 ± 36.63)	31 (215.77 ± 39.81)	0.785
Non HDL cholesterol^†^	40 (155.58 ± 35.59)	59 (153.97 ± 37.03)	0.830	19 (160.00 ± 36.41)	31 (166.16 ± 39.03)	0.581
LDL (mg/dL)^†^	40 (137.90 ± 33.04)	59 (133.90 ± 33.04)	0.523	19 (140.53 ± 31.49)	31 (146.29 ± 37.61)	0.579
HDL (mg/dL)^†^	40 (49.00 ± 1.31)	59 (49.00 ± 1.08)	0.954	19 (56.00 ± 1.95)	31 (49.00 ± 1.59)	0.203
Apo A (mg/dL)^†^	41 (0.90 ± 0.17)	58 (0.98 ± 0.16)	**0.011**	17 (1.07 ± 0.22)	29 (1.02 ± 0.19)	0.405
Apo B (mg/dL)^†^	41 (0.58 ± 0.12)	58 (0.62 ± 0.14)	0.122	17 (0.61 ± 0.15)	29 (0.68 ± 0.13)	0.105

^†^Independent sample *t*-test; and values are means ± standard deviation (SD).

^††^Mann-Whitney *U* test; and values are median ± standard error (SE).

Body mass index (BMI), waist circumference (WC), systolic blood pressure (Systolic BP), diastolic blood (Diastolic BP), pulse pressure, c-reactive protein (CRP), myeloperoxidase (MPO), nitrites, total nitric oxide metabolites (NO_*x*_), aspartate transaminase (AST), alanine transaminase (ALT), total cholesterol (t-cholesterol), non HDL cholesterol, low density lipoprotein (LDL), high density lipoprotein (HDL), Apolipoprotein A and B (Apo B and Apo A).

**Table 6:** Characterization of four distinguished groups in conformity of hypertension classification and the anthropometric, hemodynamic, cardiovascular risk biomarkers and other biochemical parameters.

	[PE > HTA] (1)	[PE > NBP] (2)	[NBPP > HTA] (3)	[NBPP > NBP] (4)	*P* value					
					(1)∗ versus (2)	(1) versus (3)	(2) versus (3)	(2) versus (4)	(3) versus (4)	(1) versus (4)
Age (years)^†^	42 (36.64 ± 5.16)	46 (33.50 ± 5.29)	6 (36.33 ± 4.97)	52 (35.50 ± 5.78)	**0.008 **	0.891	0.220	0.079	0.737	0.320
BMI (Kg/m^2^)^†^	42 (27.22 ± 5.00)	45 (26.85 ± 4.69)	6 (28.71 ± 5.11)	51 (24.97 ± 3.83)	0.830	0.500	0.373	**0.033**	**0.033**	**0.016**
WC (cm)^†^	42 (90.56 ± 18.96)	45 (88.38 ± 12.01)	6 (89.00 ± 15.11)	49 (82.06 ± 9.04)	0.516	0.848	0.908	**0.005**	0.107	**0.010**
Systolic BP (mmHg)^†^	42 (145.88 ± 16.11)	46 (125.04 ± 8.85)	6 (140.00 ± 6.23)	52 (116.44 ± 11.73)	**<0.001 **	0.384	**<0.001**	**<0.001**	**<0.001**	**<0.001**
Diastolic BP (mmHg)^†^	42 (98.90 ± 16.26)	46 (74.09 ± 10.22)	6 (86.00 ± 5.87)	52 (70.62 ± 9.23)	**<0.001 **	0.062	**0.008**	0.080	**<0.001**	**<0.001**
Pulse pressure^†^	42 (46.98 ± 14.68)	46 (50.96 ± 8.38)	6 (54.00 ± 9.53)	52 (45.83 ± 8.90)	0.144	0.263	0.413	**0.004**	**0.040**	0.658
CRP (mg/L)^††^	38 (0.60 ± 0.13)	44 (0.60 ± 0.07)	6 (0.70 ± 0.24)	49 (0.40 ± 0.12)	0.388	0.350	0.404	0.079	0.055	0.188
MPO (ng/mL)^†^	7 (82.74 ± 11.04)	25 (86.49 ± 44.39)	3 (86.83 ± 24.08)	20 (60.86 ± 29.78)	0.781	0.710	0.990	**0.032**	0.167	**0.010**
Nitrites (mol/L)^†^	7 (23.04 ± 13.07)	25 (18.02 ± 3.89)	3 (9.00 ± 0.00)	21 (9.84 ± 3.56)	0.190	0.110	**<0.001**	**<0.001**	0.693	**0.037**
NO_*x*_ (mol/L)^†^	6 (109.70 ± 40.96)	23 (96.77 ± 39.62)	3 (91.47 ± 63.01)	21 (78.63 ± 35.86)	0.474	0.610	0.839	0.120	0.600	0.134
AST (UI/L)^††^	42 (19.00 ± 0.84)	46 (20.00 ± 1.19)	6 (17.50 ± 1.25)	52 (18.00 ± 0.66)	0.589	0.190	0.188	0.108	0.564	0.230
ALT (UI/L)^††^	42 (19.00 ± 1.85)	46 (19.00 ± 2.07)	6 (15.50 ± 1.01)	52 (15.50 ± 1.17)	0.809	0.110	0.121	**0.021**	0.878	**0.031**
t-Cholesterol (mg/dL)^†^	42 (202.48 ± 42.02)	46 (210.35 ± 37.72)	6 (205.17 ± 14.10)	52 (207.48 ± 36.36)	0.267	0.878	0.739	0.701	0.879	0.538
Non HDL cholesterol^†^	42 (154.98 ± 38.86)	46 (159.80 ± 37.72)	6 (154.17 ± 15.03)	51 (158.16 ± 37.66)	0.392	0.960	0.721	0.830	0.799	0.690
LDL (mg/dL)^†^	42 (136.54 ± 37.97)	46 (137.74 ± 40.55)	6 (133.87 ± 10.29)	51 (140.25 ± 34.05)	0.632	0.866	0.818	0.741	0.326	0.690
HDL (mg/dL)^††^	42 (47.00 ± 1.29)	46 (53.00 ± 1.24)	6 (52.00 ± 3.54)	51 (50.00 ± 1.21)	0.172	0.367	0.878	0.580	0.677	0.130
Apo A (mg/dL)^†^	40 (0.97 ± 0.18)	45 (1.01 ± 0.16)	6 (0.94 ± 0.17)	50 (0.95 ± 0.21)	0.320	0.698	0.370	0.128	0.952	0.548
Apo B (mg/dL)^†^	40 (0.63 ± 0.14)	45 (0.65 ± 0.13)	6 (0.60 ± 0.06)	50 (0.59 ± 0.14)	0.704	0.584	0.387	**0.040**	0.866	0.159

^†^Independent sample *t*-test; and values are means ± standard deviation (SD); ^††^Mann-Whitney *U* test; and values are median ± standard error (SE). Relative to *P* value of (1) versus (2)∗, values were adjusted for age (regression binary logistic).

Preeclamptic women (PE); hypertension after pregnancy (HTA), normal blood pressure in pregnancy (NBPP), normotensive after pregnancy (NBP), waist circumference (WC), Body mass index (BMI), systolic blood pressure (systolic BP), diastolic blood pressure (diastolic BP), c-reactive protein (CRP), myeloperoxidase (MPO), nitrites, total nitric oxide (NO_*x*_), aspartate transaminase (AST); alanine transaminase (ALT), low density lipoprotein (LDL) and high density lipoprotein (HDL), and apolipoprotein A and B (Apo A and Apo B).

**Table 7:** Comparison of haptoglobin polymorphism between the subgroups.

	Hp 1.1 *n* (%)	Hp 2.1 *n* (%)	Hp 2.2 *n* (%)	*P* value
[PE > HTA] [1]	9 (21.4)	16 (38.1)	17 (40.5)	0.130
[PE > NBP] [2]	5 (10.9)	27 (58.7)	14 (30.4)	
[NBPP > HTA] [3]	1 (16.7)	5 (83.3)	0 (0.0)	0.185
[NBPP > NBP] [4]	9 (17.3)	25 (48.1)	18 (34.6)	

PE: Preeclamptic women; HTA: hypertension after pregnancy; NBP: normal blood pressure in pregnancy; NBP: normotensive after pregnancy.

## References

[1] Noris M, Perico N, Remuzzi G (2005). Mechanisms of disease: pre-eclampsia. *Nature Clinical Practice: Nephrology*.

[2] Intapad S, Alexander BT (2013). Future cardiovascular risk interpreting the importance of increased blood pressure during pregnancy. *Circulation*.

[3] McDonald SD, Ray J, Teo K (2013). Measures of cardiovascular risk and subclinical atherosclerosis in a cohort of women with a remote history of preeclampsia. *Atherosclerosis*.

[4] Staff AC (2011). Circulating predictive biomarkers in preeclampsia. *Pregnancy Hypertension*.

[5] Chen CW, Jaffe IZ, Karumanchi SA (2014). Pre-eclampsia and cardiovascular disease. *Cardiovascular Research*.

[6] Langlois MR, Delanghe JR (1996). Biological and clinical significance of haptoglobin polymorphism in humans. *Clinical Chemistry*.

[7] Levy AP, Asleh R, Blum S (2010). Haptoglobin: basic and clinical aspects. *Antioxidants and Redox Signaling*.

[8] Theilgaard-Mönch K, Jacobsen LC, Nielsen MJ (2006). Haptoglobin is synthesized during granulocyte differentiation, stored in specific granules, and released by neutrophils in response to activation. *Blood*.

[9] Akila P, Prashant V, Suma MN, Prashant SN, Chaitra TR (2012). CD163 and its expanding functional repertoire. *Clinica Chimica Acta*.

[10] Graversen JH, Madsen M, Moestrup SK (2002). CD163: a signal receptor scavenging haptoglobin-hemoglobin complexes from plasma. *International Journal of Biochemistry and Cell Biology*.

[11] Nielsen MJ, Møller HJ, Moestrup SK (2010). Hemoglobin and heme scavenger receptors. *Antioxidants and Redox Signaling*.

[12] Gruys E, Toussaint MJM, Niewold TA, Koopmans SJ (2005). Acute phase reaction and acute phase proteins. *Journal of Zhejiang University: Science*.

[13] Vallelian F, Schaer CA, Kaempfer T (2010). Glucocorticoid treatment skews human monocyte differentiation into a hemoglobin-clearance phenotype with enhanced heme-iron recycling and antioxidant capacity. *Blood*.

[14] Van Vlierberghe H, Langlois M, Delanghe J (2004). Haptoglobin polymorphisms and iron homeostasis in health and in disease. *Clinica Chimica Acta*.

[15] Azarov I, He X, Jeffers A (2008). Rate of nitric oxide scavenging by hemoglobin bound to haptoglobin. *Nitric Oxide—Biology and Chemistry*.

[16] Alayash AI (2011). Haptoglobin: old protein with new functions. *Clinica Chimica Acta*.

[17] Kendall PA, Saeed SA, Collier HOJ (1979). Identification of endogenous inhibitor of prostaglandin synthetase with haptoglobin and albumin. *Biochemical Society Transactions*.

[18] Saeed SA, Ahmad N, Ahmed S (2007). Dual inhibition of cyclooxygenase and lipoxygenase by human haptoglobin: Its polymorphism and relation to hemoglobin binding. *Biochemical and Biophysical Research Communications*.

[19] Cid MC, Grant DS, Hoffman GS, Auerbach R, Fauci AS, Kleinman HK (1993). Identification of haptoglobin as an angiogenic factor in sera from patients with systemic vasculitis. *Journal of Clinical Investigation*.

[20] Guetta J, Strauss M, Levy NS, Fahoum L, Levy AP (2007). Haptoglobin genotype modulates the balance of Th1/Th2 cytokines produced by macrophages exposed to free hemoglobin. *Atherosclerosis*.

[21] Asleh R, Miller-Lotan R, Aviram M (2006). Haptoglobin genotype is a regulator of reverse cholesterol transport in diabetes in vitro and in vivo. *Circulation Research*.

[22] Brown MA, Lindheimer MD, de Swiet M, van Assche A, Moutquin JM (2001). The classification and diagnosis of the hypertensive disorders of pregnancy: statement from the International Society for the Study of Hypertension in Pregnancy (ISSHP). *Hypertension in Pregnancy*.

[23] (2013). 2013 Practice guidelines for the management of arterial hypertension of the European society of hypertension (ESH) and the European society of cardiology (ESC): ESH/ESC task force for the management of arterial hypertension. *Journal of Hypertension*.

[24] Linke RP (1984). Typing and subtyping of haptoglobin from native serum using disc gel electrophoresis in alkaline buffer: application to routine screening. *Analytical Biochemistry*.

[25] Guerra A, Monteiro C, Breitenfeld L (1997). Genetic and environmental factors regulating blood pressure in childhood: prospective study from 0 to 3 years. *Journal of Human Hypertension*.

[26] Highton J, Hessian P (1984). A solid-phase enzyme immunoassay for C-reactive protein: clinical value and the effect of rheumatoid factor. *Journal of Immunological Methods*.

[27] Valenzuela FJ, Pérez-Sepúlveda A, Torres MJ, Correa P, Repetto GM, Illanes SE (2012). Pathogenesis of preeclampsia: the genetic component. *Journal of Pregnancy*.

[28] Cooke CM, Brockelsby JC, Baker PN, Davidge ST (2003). The Receptor for Advanced Glycation End Products (RAGE) is elevated in women with preeclampsia. *Hypertension in Pregnancy*.

[29] Huang QT, Zhang M, Zhong M (2013). Advanced glycation end products as an upstream molecule triggers ROS-induced sFlt-1 production in extravillous trophoblasts: a novel bridge between oxidative stress and preeclampsia. *Placenta*.

[30] Bicho MC, da Silva AP, Medeiros R, Bicho M, Janciauskiene S (2013). The role of haptoglobin and its genetic polymorphism in cancer: a review. *Acute Phase Proteins*.

[31] Sammour RN, Nakhoul FM, Levy AP (2010). Haptoglobin phenotype in women with preeclampsia. *Endocrine*.

[32] Weissgerber TL, Gandley RE, McGee PL (2013). Haptoglobin phenotype, preeclampsia risk and the efficacy of vitamin C and E supplementation to prevent preeclampsia in a racially diverse population. *PLoS ONE*.

[33] Weissgerber TL, Roberts JM, Jeyabalan A (2012). Haptoglobin phenotype, angiogenic factors, and preeclampsia risk. *The American Journal of Obstetrics and Gynecology*.

[34] Berkova N, Lemay A, Dresser DW, Fontaine J, Kerizit J, Goupil S (2001). Haptoglobin is present in human endometrium and shows elevated levels in the decidua during pregnancy. *Molecular Human Reproduction*.

[35] Gloria-Bottini F, Bottini N, La Torre M, Magrini A, Bergamaschi A, Bottini E (2008). The effects of genetic and seasonal factors on reproductive success. *Fertility and Sterility*.

[36] Depypere HT, Langlois MR, Delanghe JR, Temmerman M, Dhont M (2006). Haptoglobin polymorphism in patients with preeclampsia. *Clinical Chemistry and Laboratory Medicine*.

[37] Raijmakers MT, Roes EM, Te Morsche RH, Steegers EA, Peters WH (2003). Haptoglobin and its association with the HELLP syndrome. *Journal of Medical Genetics*.

[38] McElrath TF, Lim K, Pare E (2012). Longitudinal evaluation of predictive value for preeclampsia of circulating angiogenic factors through pregnancy. *American Journal of Obstetrics and Gynecology*.

[39] Baldus S, Heitzer T, Eiserich JP (2004). Myeloperoxidase enhances nitric oxide catabolism during myocardial ischemia and reperfusion. *Free Radical Biology and Medicine*.

[40] Baldus S, Rudolph V, Roiss M (2006). Heparins increase endothelial nitric oxide bioavailability by liberating vessel-immobilized myeloperoxidase. *Circulation*.

