# Supplementary material for: In Women with Previous Pregnancy Hypertension, Levels of Cardiovascular Risk Biomarkers May Be Modulated by Haptoglobin Polymorphism
Source: Obstet Gynecol Int. 2014 Jul 2;2014:361727. doi: 10.1155/2014/361727 (PMC4102073; doi:10.1155/2014/361727)
Supplement: Supplementary file 1 — In supplementary table, four distinguishes groups, which results from women that were classified in preeclamptic (PE) and normal blood pressure in pregnancy (NBPP) and reclassified in hypertensive after pregnancy (HTA) and normotensive after pregnancy (NBP), based on the criteria of European Society of Hypertension (ESH) and European Society of Cardiology (ESC). These groups were characterized in conformity of hypertension classification and anthropometric, hemodynamic, cardiovascular risk biomarkers and other biochemical parameters in Hp 1.1 plus 2.1 phenotypes. [file 361727.f1.pdf]

***Supplementary table: Characterization of four distinguish groups in conformity of hypertension classification and the anthropometric, hemodynamic, cardiovascular risk biomarkers and other biochemical parameters in* Hp 1.1 plus 2.1 phenotypes.**

	[PE > HTA] (1)	[PE > NBP] (2)	[NBPP > HTA] (3)	[NBPP > NBP] (4)	P value 1.1 plus 2.1					
					(1) *Versus* (2)	(1) *Versus* (3)	(2) *Versus* (3)	(2) *Versus* (4)	(3) *Versus* (4)	(1) *Versus* (4)
**Age (years)**	25 (36.60 ± 4.56)	32 (33.66 ± 5.62)	6 (36.33 ± 4.97)	34 (35.50 ± 5.81)	**0.038**	0.900	0.284	0.195	0.743	0.435
**BMI (Kg/m²) †**	25 (26.97 ± 5.93)	31 (26.88 ± 5.23)	6 (28.71 ± 5.11)	34 (24.45 ± 3.22)	0.954	0.514	0.437	**0.026**	**0.010**	0.063
**WC (cm) †**	25 (92.74 ± 22.68)	31 (88.94 ± 12.51)	6 (89.00 ± 15.11)	32 (81.20 ± 8.47)	0.429	0.706	0.991	**0.005**	0.078	**0.022**
**Systolic BP (mmHg) †**	25 (147.56 ± 19.17)	32 (124.56 ± 9.02)	6 (140.00 ± 6.23)	34 (115.50 ± 11.06)	**< 0.001***	0.353	**< 0.001**	**0.001**	**< 0.001**	**< 0.001**
**Diastolic BP (mmHg) †**	25 (100.92 ± 18.48)	32 (74.69 ± 10.26)	6 (86.00 ± 5.87)	34 (70.47 ± 10.26)	**< 0.001***	0.063	**0.013**	0.100	**0.001**	**< 0.001**
**Pulse pressure †**	25 (46.64 ± 15.13)	32 (49.88 ± 8.56)	6 (54.00 ± 9.53)	32 (45.03 ± 7.19)	0.312	0.268	0.294	**0.015**	**0.011**	0.625
**CRP (mg/L) ††**	24 (0.45 ± 0.19)	30 (0.50 ± 0.09)	6 (0.70 ± 0.24)	32 (0.30 ± 0.17)	0.713	0.112	0.201	0.217	**0.029**	0.488
**MPO (ng/mL) †**	3 (80.33 ± 6.42)	15 (96.93 ± 45.84)	3 (86.83 ± 24.08)	13 (54.38 ± 30.75)	0.550	0.675	0.720	**0.009**	0.112	**0.014**
**Nitrites (µmol/L) †**	3 (30.40 ± 19.11)	15 (17.90 ± 2.89)	3 (9.00 ± 0.00)	14 (8.99 ± 2.32)	0.375	0.192	**< 0.001**	**< 0.001**	0.992	0.191
**NOx (µmol/L) †**	3 (111.93 ± 62.36)	15 (99.04 ± 47.96)	3 (91.47 ± 63.00)	14 (78.19 ± 33.30)	0.692	0.710	0.816	0.194	0.597	0.188
**AST (UI/L) †**	25 (19.16 ± 4.63)	32 (20.44 ± 5.52)	6 (17.17 ± 3.06)	34 (18.32 ± 4.72)	0.357	0.327	0.170	0.099	0.568	0.500
**ALT (UI/L) ††**	25 (18.00 ± 1.23)	32 (18.00 ± 1.65)	6 (15.50 ± 1.01)	34 (15.00 ± 1.34)	0.729	0.227	0.185	**0.025**	0.761	0.055
**t-Cholesterol (mg/dL) †**	25 (194.36 ± 41.45)	32 (207.25 ± 36.41)	6 (205.17 ± 14.09)	34 (203.74 ± 36.14)	0.217	0.294	0.892	0.695	0.925	0.359
**Non HDL cholesterol †**	25 (147.96 ± 37.72)	32 (156.56 ± 36.81)	6 (154.17 ± 15.03)	33 (155.97 ± 38.82)	0.390	0.698	0.877	0.950	0.912	0.434
**HDL (mg/dL) †**	25 (46.40 ± 8.46)	32 (50.69 ± 7.85)	6 (51.00± 8.67)	33 (48.27 ± 8.39)	**0.053**	0.243	0.930	0.235	0.470	0.405
**LDL (mg/dL) †**	25 (129.73 ± 37.77)	32 (134.14 ± 41.03)	6 (133.87 ± 10.29)	33 (138.87 ± 36.18)	0.679	0.794	0.974	0.630	0.745	0.358
**Apo A (mg/dL) †**	25 (0.96 ± 0.18)	31 (0.99 ± 0.14)	6 (0.94 ± 0.17)	34 (0.89 ± 0.17)	0.424	0.841	0.445	**0.011**	0.495	0.142
**Apo B (mg/dL) †**	25 (0.60 ± 0.14)	32 (0.63 ± 0.14)	6 (0.69 ± 0.06)	34 (0.58 ± 0.13)	0.546	0.870	0.411	0.126	0.679	0.422

† Independent sample T – test; and values are means ± standard deviation (SD); †† Mann – Whitney U test; and values are median ± standard error (SE); Relatively to p value of (1) versus (2)*, values were adjusted for age (regression binary logistic).

Preeclamptic women (PE); hypertension after pregnancy (HTA); normal blood pressure in pregnancy *(NBPP); normotensive after pregnancy (NBP); waist circumference (WC), Body mass index (BMI), systolic blood pressure (Systolic BP), diastolic blood pressure (Diastolic BP), c – reactive protein (CRP), myeloperoxidase (MPO), nitrites, total nitric oxide (NOx), aspartate transaminase (AST); alanine transaminase (ALT), low density lipoprotein (LDL) and high density lipoprotein (HDL), Apolipoprotein A and B (Apo A and Apo B).*
